# Fabrication of Helical Carbon Fiber Skeleton Using Arc Glow Discharge Method

**DOI:** 10.3390/ma17174181

**Published:** 2024-08-23

**Authors:** Xiye Chen, Haiyong Chen, Yongjun Bao, Yuhan Meng, Zhigang Jiang

**Affiliations:** State Key Laboratory of Superhard Materials, College of Physics, Jilin University, Changchun 130012, China; chenxy@chenhc.cn (X.C.); yjbao@jlu.edu.cn (Y.B.); jiangzg@jlu.edu.cn (Z.J.)

**Keywords:** arc glow discharge, helical carbon fiber, helical carbon fiber skeleton

## Abstract

An arc glow discharge device was used to prepare a helical carbon fiber skeleton with helical carbon fibers hooked to each other by spraying a hydrogen and ethanol mixture onto the iron wire substrate through the discharge area, using anhydrous ethanol as the carbon source. The samples were characterized by SEM, EDS, Raman and XPS. A growth mechanism of helical carbon fiber driven by C sp3 was proposed. The various growth modes of carbon fiber during the formation of carbon fiber skeleton were investigated. A ring appearance that indicated a change in the direction of carbon fiber growth was observed. And double helical carbon fiber was constructed from single helical carbon fiber in two ways. Super-large carbon fiber with a diameter of about 13 μm was observed, and it was speculated that this super-large carbon fiber is the backbone of the carbon fiber skeleton. The mechanical properties of the carbon fiber skeleton are isotropic.

## 1. Introduction

Helical carbon fiber possesses distinctive mechanical [1] and electrical [2] properties, which can be used in wave-absorbing materials [3], hydrogen storage materials [4], electrode materials [5], reinforced composite materials [6] and so on. Consequently, it exhibits a vast range of potential applications. However, the helical carbon fiber is faced with the problems of low output, difficult mass preparation and high price. Therefore, it is important to clarify the formation mechanism of helical carbon fiber and develop the growth method of helical carbon fiber with high yield. Helical carbon fiber was first obtained in 1953 by using iron oxide as a catalyst to crack carbon monoxide [7]. Baker [8] et al. chose to use acetylene as a carbon source and successfully prepared carbon fiber with a helical structure using an alloy catalyst. Motojima et al. [9] used chemical vapor deposition, using Ni powder as a catalyst and acetylene as a carbon source to prepare regular double helical carbon fibers at temperatures between 350 and 75 °C. The preparation methods and growth conditions of helical carbon fibers have been consistently updated and refined since then. In this study, we present a simplified approach to achieve the growth conditions necessary for the synthesis of helical carbon fibers. By utilizing anhydrous ethanol as the carbon source and iron wire as the growth substrate, we successfully fabricated a helical carbon fiber skeleton formed by a large number of helical carbon fibers hooking with each other.

## 2. Materials and Methods

**Figure 1:**
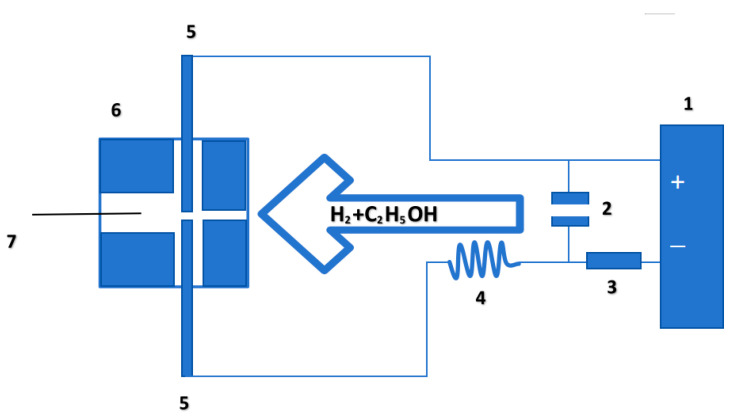
Experimental instrument diagram. (1) DC power supply; (2) capacitance; (3) resistance; (4) inductance; (5) Tungsten needle electrode; (6) graphite cavity; (7) iron wire.

Using the arc glow discharge device as shown in Figure 1 [10], graphite is selected as the growth cavity, iron wire is selected as the substrate, arc glow discharge is carried out between two tungsten needle electrodes, and the mixed gas of ethanol and hydrogen passes through the discharge area, becomes activated particles, and then is sprayed onto the wire substrate to realize the growth of helical carbon fiber; the entire growth process occurs under atmospheric pressure.

The graphite cavity is a 50 × 50 × 20 mm square with 4 holes inside. Two holes are used to house the tungsten needle electrode; one hole with a diameter of 2 mm is used to pass the gas mixture, and the other hole with a diameter of 10 mm is used to place the iron wire. The distance between the wire and the tungsten needle electrode is 10 mm. Hydrogen is prepared by a hydrogen generator at a flow rate of 500 mL per minute and then passed into an alcohol bottle to obtain a mixed gas of alcohol and hydrogen.

The experimental parameters encompass a capacitance of 0.1 μF, an inductance of 0.4 mH, a current-limiting resistance of 150 Ω and a voltage of 550 V, and the discharge power is determined using the following formula:

P = UI − RI^2^ (1)

where P represents power, U represents the voltage of the DC power supply, and I represents electric current.

In this experiment, the current is controlled by adjusting only the distance between the two tungsten needle electrodes, while keeping other experimental parameters such as gas flow rate, capacitance, inductance, resistance and voltage unchanged. The greater the distance, the smaller the corresponding current. 

In the experiment, the control currents were set at 2.3 A, 2.4 A, 2.5 A, 2.7 A and 2.9 A, resulting in corresponding power outputs of 483 W, 468 W, 450 W, 405 W and 348 W.

The graphite cavity was preheated for a duration of 10 min, followed by a growth period of 30 min on the wire substrate. The black sample was found to be almost full of growth holes with a diameter of 10 mm.

The substrate material was replaced by replacing the iron wire substrate with Fe-Cr-Al wire and a tungsten needle. Under identical growth conditions, no discernible growth marks were observed on samples grown using Fe-Cr-Al wire and tungsten needle.

## 3. Results 

### 3.1. Materials Characterization

**Figure 2:**
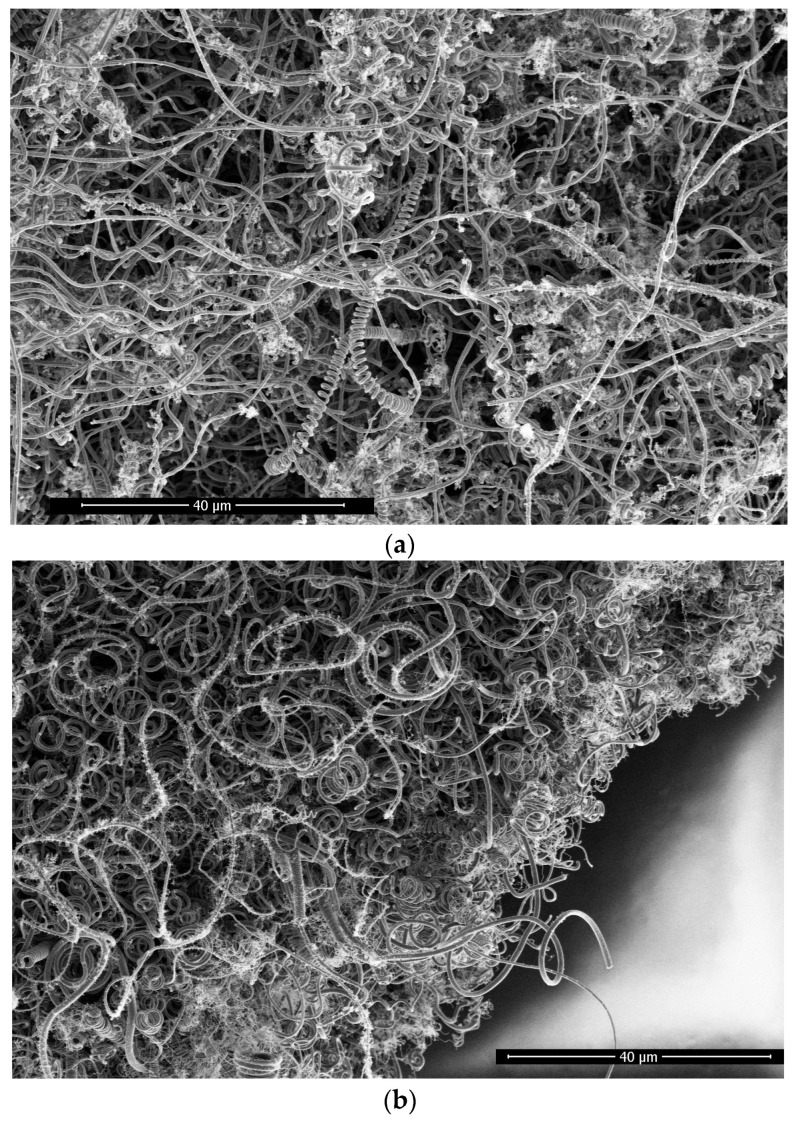

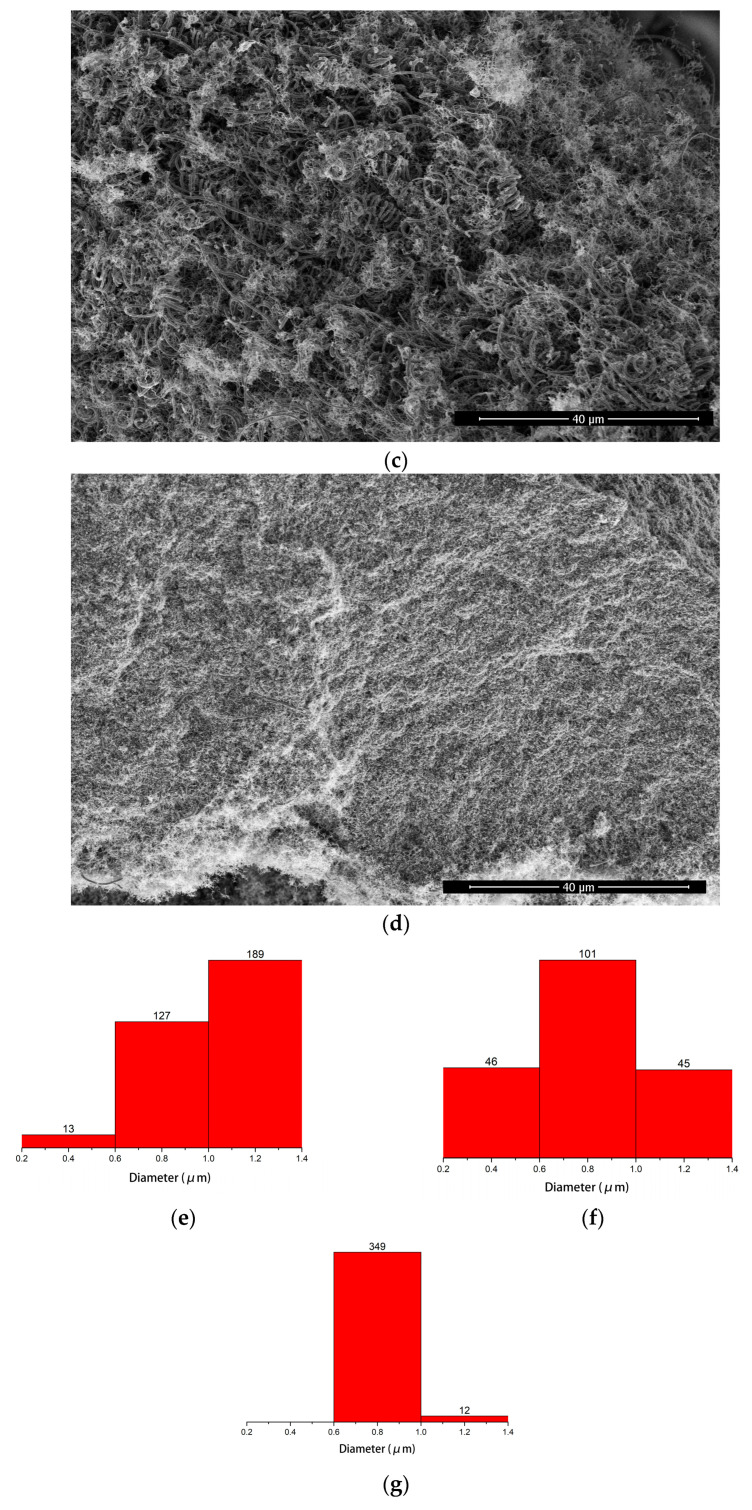
(**a**) The growth current of the sample is 2.3 A; (**b**) the growth current of the sample is 2.5 A; (**c**) the growth current of the sample is 2.7 A; (**d**) the growth current of the sample is 2.9 A; (**e**) statistical diagram of the fiber diameter of the sample under growth conditions of the 2.3 A current; (**f**) statistical diagram of the fiber diameter of the sample under growth conditions of the 2.5 A current; (**g**) statistical diagram of the fiber diameter of the sample under growth conditions of the 2.7 A current.

A Magellan 400 field emission scanning electron microscope was used to scan samples obtained on an iron wire substrate using the arc glow discharge method. The control currents of samples were set at 2.3 A, 2.5 A, 2.7 A and 2.9 A. As shown in Figure 2a–d, the sample was found to be a carbon fiber with a helical structure. The fiber diameter statistics were carried out for samples with a growth condition of current 2.3 A, as shown in Figure 2e. Among them, there were 13 carbon fibers with sizes of about 0.4 μm, 127 fibers with sizes of about 0.8 μm, and 189 fibers with sizes of about 1.2 μm. It was found that the number of fibers with a size of about 1.2 μm was the largest. Fiber diameter statistics were performed for the sample with a growth condition of current 2.5 A, as shown in Figure 2f. In this case, there were 46 carbon fibers with sizes around 0.4 μm, 101 fibers with sizes around 0.8 μm, and 45 fibers with sizes around 1.2 μm. The largest number of fibers had a size around 0.8 μm. Fiber diameter statistics were conducted for the sample with a growth condition of current 2.7 A, as shown in Figure 2g. Within the range of 0.8 to 1.2 μm, there were 349 and 12 carbon fibers counted, respectively. Due to the large number of tiny carbon fibers smaller than 0.4 μm, they were not included in the count. According to the statistics, as current decreases, the diameter of carbon fiber increases.

In the sample diagram, with a growth current of 2.9 A, tiny carbon fibers with a diameter of less than 0.4 μm covered almost the entirety of Figure 2d, and occasionally, carbon fibers with a diameter greater than 0. 4 μm were observed. According to Figure 2a–d, we could also see that as the current increased, the number of tiny carbon fibers also increased, indicating that a larger current promotes the growth of tiny carbon fibers.

**Figure 3:**
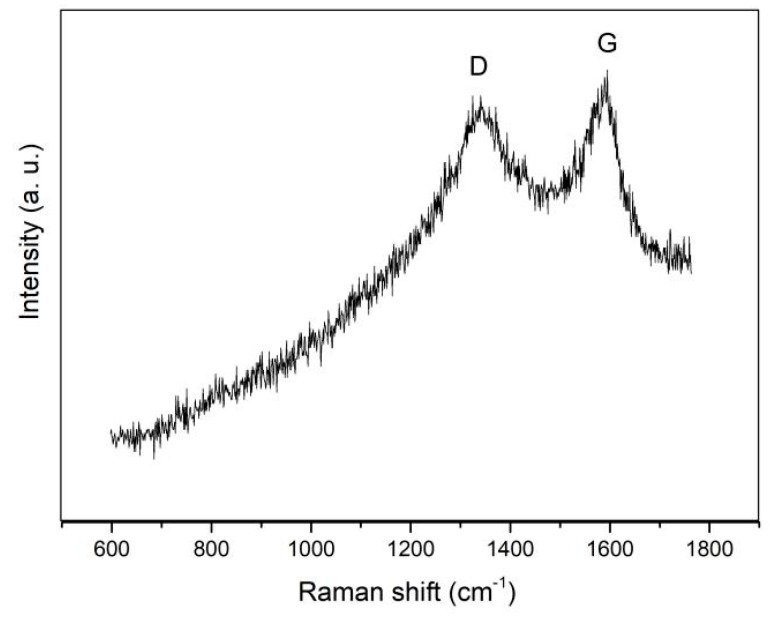
Raman spectrum of helical carbon fiber sample. The G peak represents the stretch vibration of C=C bonds in the sp^2^ structure, and the D peak corresponds to the breath vibration mode of hexatomic rings which appears when defects exist.

The Raman spectrum of the helical carbon fiber is shown in Figure 3, with an excitation wavelength of 532 nm. The D peak around 1340 cm^−1^ and the G peak around 1591 cm^−1^ are two characteristic peaks for graphite materials. The intensities of the D peak and G peak were similar, indicating the presence of numerous defects in the helical carbon fiber.

**Figure 4:**
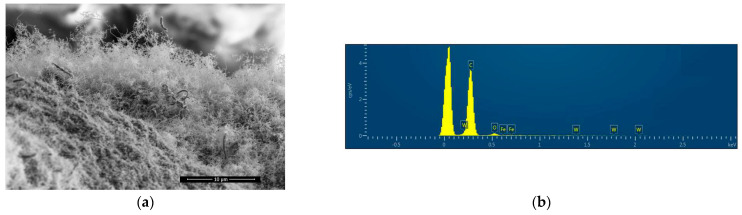
(**a**) The sample under the growth current of 2.9 A; (**b**) Energy Dispersive Spectrum of the sample.

As shown in Figure 4a, the helical carbon fiber, for which the growth current of the sample is 2.9 A, was covered by tiny carbon fiber wraps. Area scanning was performed to obtain the Energy Dispersive Spectrum of the sample, the selected area was the entirety of Figure 4a. The presence of C and O peaks was observed in Figure 4b, while Fe and W peaks were absent, indicating the absence of both Fe and W in the sample. This suggests that there was no diffusion of iron wire substrate into the carbon fiber sample, and the tungsten needle electrode was not sprayed onto the sample.

**Figure 5:**
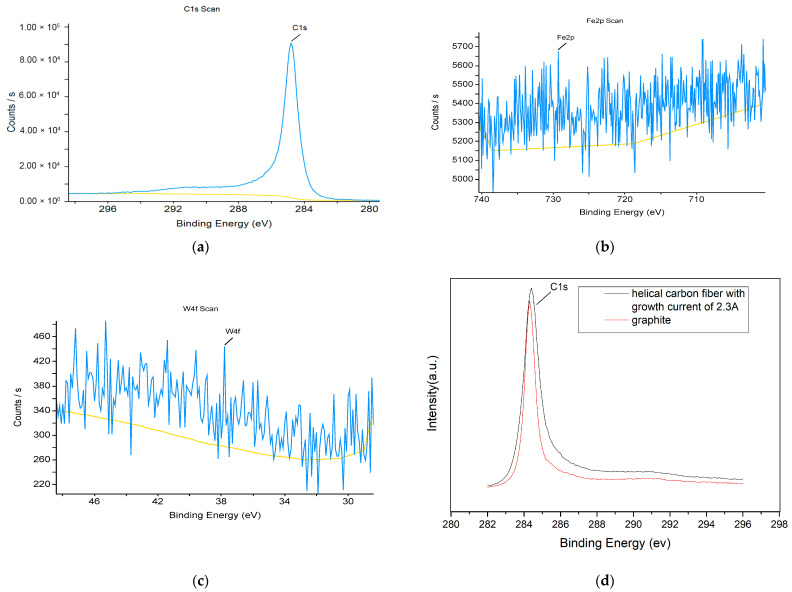
X-ray photoelectron spectra and peak binding energy of helical carbon fiber sample with current of 2.3A. (**a**) C1s; (**b**) Fe2p; (**c**) W4f; Blue line is experimental data, yellow line is fiting base line. (**d**) C1s peaks of graphite and helical carbon fiber.

The surface chemical composition and valence state of helical carbon fiber with growth current of 2.3 A were determined by XPS measurement. Compounds were identified by corresponding binding energy peaks. XPS analysis was performed using Avantage software (v5). The XPS spectrum is shown in Figure 5a–c, and the measured spectra were C1s, Fe2p and W4f. The fitting atom percentages of carbon, iron and tungsten at the surface of the helical carbon fiber were 99.56%, 0.39% and 0.05%. The data show that the helical carbon fiber obviously did not contain iron and tungsten. As shown in Figure 5d, the XPS pattern of the sample was compared with that of graphite, and it was found that the C1s peak of graphite was 284.3 ev with a half-peak width of 0.6 ev, while the C1s peak of helical carbon fiber was 284.4 ev with a half-peak width of 0.9 ev. This shows that the binding energy peak of the sample broadened towards high energy. The fitting results of the XPS C1s peak in the diamond-like carbon film showed a peak at 284.3 ev for C sp^2^ and a higher peak at 295.3 ev for C sp^3^, indicating that the binding energy of C sp^3^ was greater than that of C sp^2^ [11]. We believe that the peak shift of the sample was due to the incorporation of C sp^3^ into the graphite layer as an impurity.

### 3.2. Helical Carbon Fiber

#### 3.2.1. Longitudinal Growth Splitting Mode 

Upon observation of the helical carbon fiber samples, it was discovered that the carbon fibers exhibited three distinct structural types: single-wire, double-wire and a transitional line structure featuring an intermediate dent.

**Figure 6:**
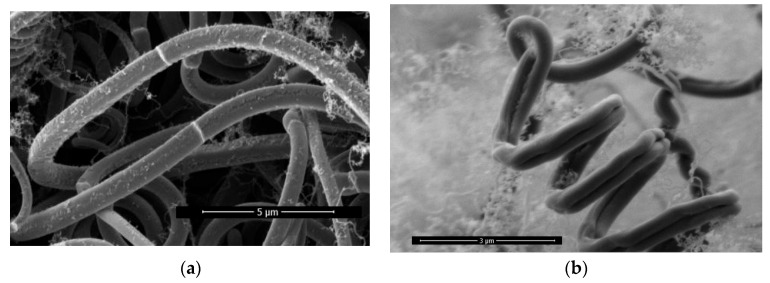

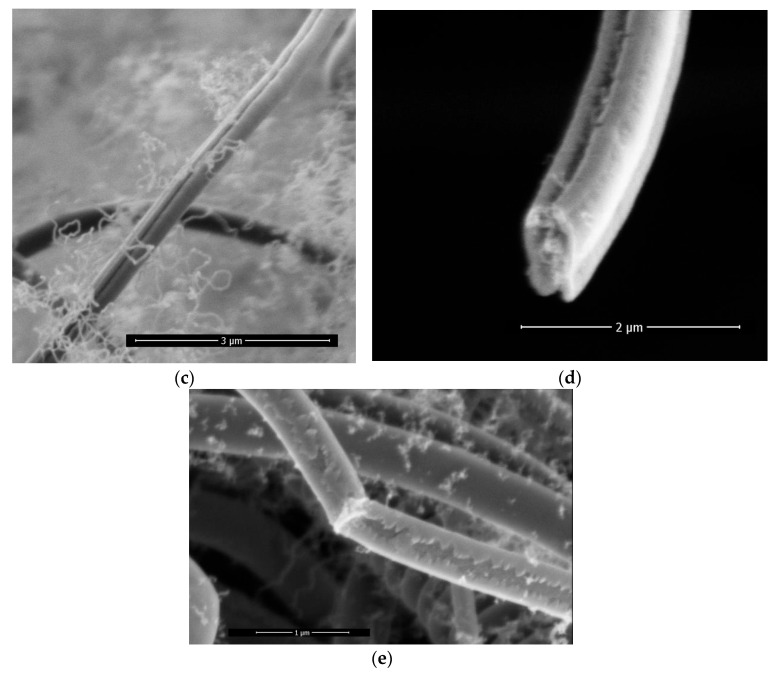
(**a**) Two single-wire carbon fibers; (**b**) double-wire helical structure; (**c**) carbon fiber with an intermediate dent; (**d**) cross-section of the carbon fiber of the transition line; (**e**) a very shallow intermediate dent on the surface of a single-wire carbon fiber.

Figure 6a shows a partial photo of a carbon fiber sample grown at a current of 2.5 A. The surface of the two single-wire carbon fibers in the middle is smooth and uniform in diameter, showing an obvious single-wire structure. 

The diagram in Figure 6b represents the sample under current condition 2.3 A; the carbon fibers that make up this helical structure have significant intermediate dents in the middle, so we believe it is a double-wire helical structure. Double-wire opening occurs at the end of a double-wire helical carbon fiber. The two carbon fibers grow in different directions.

Figure 6c shows a carbon fiber under current condition 2.3 A with an intermediate dent; however, since the dent is not deep enough to split into two single-wire carbon fibers, it is identified as a transitional line form of carbon fiber.

Figure 6d shows the cross-section of the carbon fiber of the transition line, where both the upper and lower sides of the dent marks can be observed. The growth current of the sample is 2.5 A.

The single-wire carbon fiber under current condition 2.5 A, as shown in Figure 6e, exhibits a very shallow intermediate dent on the surface. We believe this indicates the initial stages of carbon fiber transformation.

There is a longitudinal growth splitting mode of carbon fiber; single-wire carbon fiber will evolve intermediate dents in the process of longitudinal growth, and as the degree of dents deepens, it will become synchronous with the growth of double-wire carbon fiber. Under certain conditions, the double-wire carbon fiber opens the double-wire structure and completes the splitting phenomenon from one carbon fiber to two carbon fibers.

#### 3.2.2. Secondary Nucleation Growth

**Figure 7:**
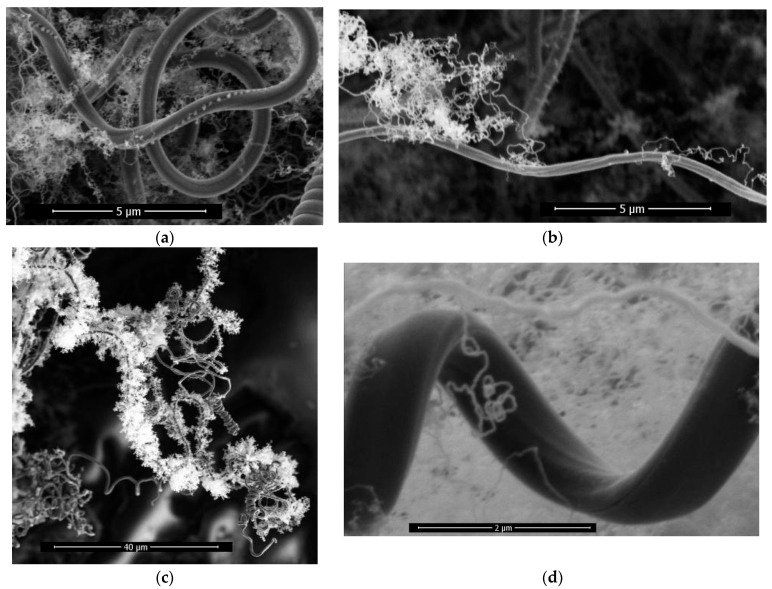
(**a**) Nuclei are arranged in the intermediate dent of the transition line structure; (**b**) a small number of tiny carbon fibers grow on the side wall of a single carbon fiber; (**c**) the side walls of a single carbon fiber are completely covered by tiny carbon fibers; (**d**) size comparison of carbon fiber and tiny carbon fibers.

As shown in Figure 7a, the growth current condition of the sample is 2.3 A, and it is observed that nuclei are arranged in the intermediate dent of the transition line structure. In Figure 7b, a small number of tiny carbon fibers grow on the side wall of a single carbon fiber, for which the growth current condition is 2.3 A. In Figure 7c, the growth current condition of the sample is 2.3 A, and the side walls of a single carbon fiber are completely covered by tiny carbon fibers.

The helical carbon fiber can be observed as a result of secondary nucleation growth, involving two distinct stages of nucleation and linear growth. Furthermore, it demonstrates the excellent suitability of carbon fiber as a self-nucleation substrate. This phenomenon of the growth of tiny carbon fibers after the side-wall nucleation of carbon fibers is called the secondary nucleation growth phenomenon of carbon fibers.

The diameter of the tiny carbon fiber obtained by secondary nucleation growth is much smaller than that of the substrate carbon fiber, as shown in Figure 7d, the diameters of which are about 0.8 μm for the substrate carbon fiber and 0.03 μm for the secondary nucleation growth carbon fiber; the growth current condition of the sample is 2.3 A.

The tiny carbon fiber obtained by secondary nucleation growth on the side wall of the carbon fiber can occupy the void as much as possible in the limited space, increasing the surface area of the carbon fiber per unit volume.

#### 3.2.3. Change-Direction Ring

**Figure 8:**
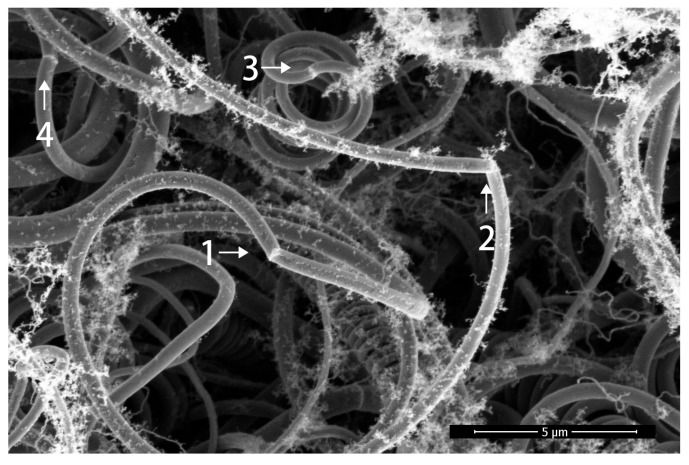
Change-direction ring indicates that the growth direction of carbon fiber has changed. Arrows 1, 2, 3 and 4 indicate change-direction rings.

As shown in Figure 8, there is a ring appearance on a single-wire carbon fiber; the growth current condition is 2.5 A. With this ring as the dividing line, the growth direction of carbon fiber changes, and the curvature radius and fiber diameter at both sides of the ring basically do not change. This ring appearance of carbon fiber only indicates that the growth direction of carbon fiber has changed, which is called the change-direction ring. 

#### 3.2.4. Constructing Double Helical Carbon Fibers

**Figure 9:**
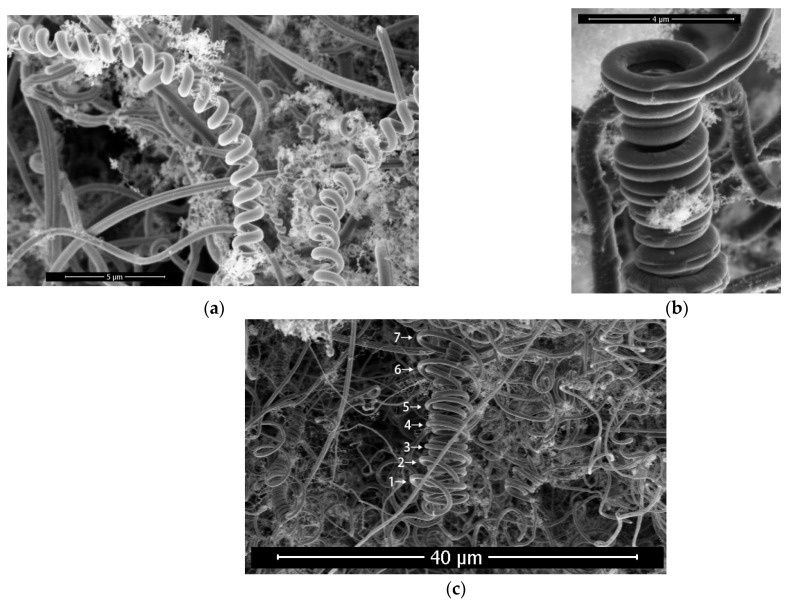
(**a**) Single helical carbon fiber; (**b**) double helical carbon fiber is formed by double-wire carbon fiber; (**c**) single helical carbon fiber round trip to form double helical carbon fiber. Arrows indicate different helical circles, while the numbers represent their order.

As shown in Figure 9a, the fiber diameter of the single helical carbon fiber is about 0.6 μm, the pitch is about 1.1 μm and the helical diameter is about 1.5 μm, where the growth current of the sample is 2.3 A. Figure 9b,c show two different ways of constructing double helical carbon fibers; both samples were obtained under the growth current of 2.3 A. The double helical carbon fiber in Figure 9b is formed by the double-wire carbon fiber. In Figure 9c, there is a change-direction ring at the top of the helical carbon fiber of the sample. After the helical carbon fiber has experienced the change-direction ring, the first and second helical loops of subsequent growth are inserted into the interior of the original helical structure, the third, fourth and fifth helical loops are extended outside the original helical structure and the helical radius of the sixth and subsequent loops numbers is gradually increased. This indicates that the helical structure after the change-direction ring is influenced by the helical structure prior to the change-direction ring. It is shown that the change-direction ring is not only the end point of the original helical carbon fiber, but also the beginning of the new helical carbon fiber. The new helical carbon fiber and the original helical carbon fiber form a double helical structure together.

### 3.3. Helical Carbon Fiber Skeleton

**Figure 10:**
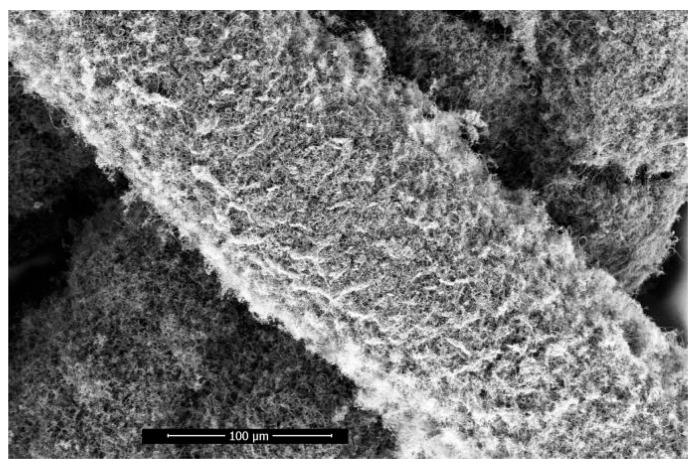
Helical carbon fiber skeleton.

The longitudinal growth splitting mode of carbon fiber and the secondary nucleation growth mode of the side wall can be carried out simultaneously, and the initial nucleation position can be spread over any side wall of carbon fiber, which has randomness. Moreover, the direction of the helical growth of carbon fiber after the change-direction ring has randomness, and finally, the distribution of helical carbon fiber in space has randomness. In a specific space, this random growth characteristic results in the hooking of helical carbon fibers with other helical carbon fibers. As shown in Figure 10, with increasing growth time, the helical carbon fiber eventually grows to form a carbon fiber skeleton; the growth current condition of the sample is 2.7 A. Furthermore, helical carbon fiber skeletons are observed in all samples when the current range falls between 2.3 and 2.9 A.

#### 3.3.1. Super-Large Carbon Fiber

**Figure 11:**
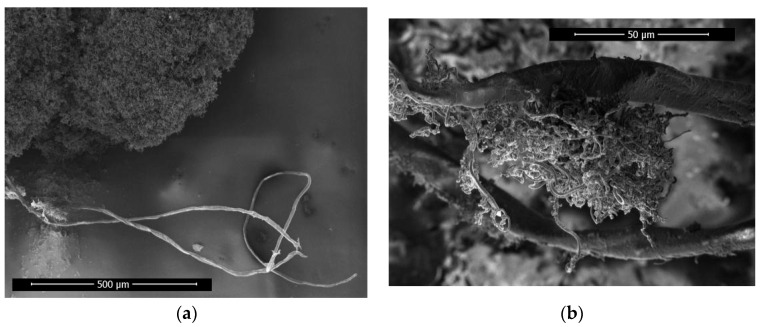
(**a**) Super-large carbon fiber overall appearance; (**b**) super-large carbon fiber and common carbon fiber interaction diagram.

In Figure 11a, the growth current condition of the sample is 2.4 A, and super-large carbon fiber with a diameter of about 13 μm is found in the sample, which is much larger than ordinary carbon fiber. The diameter of the super-large carbon fiber is equivalent to the helical diameter of the ordinary helical carbon fiber and visible to the human eye. Therefore, we designated it as “super-large carbon fiber”.

As shown in Figure 11b, carbon fibers with a diameter of approximately 1.3 μm are grown on the super-large carbon fiber; the growth current condition of the sample is 2.4 A. The 1.3 μm carbon fiber is considered as the product of secondary nucleation growth from the super-large carbon fiber.

Moreover, the super-large carbon fiber length is of the same order of magnitude as the length of the carbon fiber skeleton in the sample, suggesting that the super-large fiber serves as a core component for priority growth within the carbon fiber skeleton, which acts as its backbone.

#### 3.3.2. The Torn State of the Helical Carbon Fiber Skeleton

When the helical carbon fiber skeleton is subjected to external force, it will resist the external force in the form of internal carbon fiber deformation.

**Figure 12:**
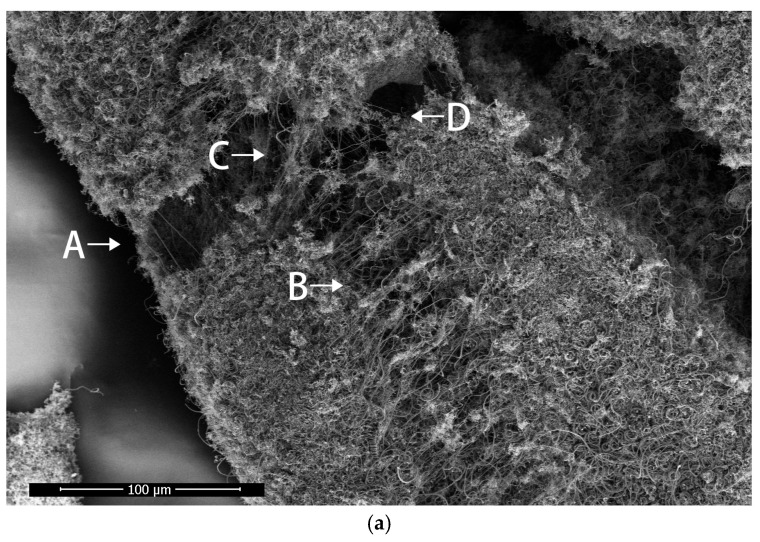

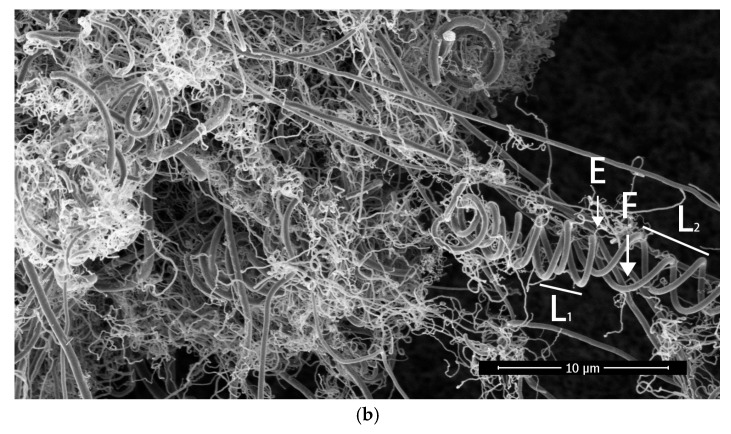
(**a**) The torn state of the helical carbon fiber skeleton, the carbon fibers are stretched at A, B, C and D; (**b**) a nearly straight carbon fiber passes through the middle of a broken double helical carbon fiber to form a hooked structure. The contact points are E and F, L_1_ and L_2_ show the pitchs.

Figure 12a is a tear diagram of the helical carbon fiber skeleton; the growth current condition of the sample is 2.7 A. It can be observed that there are carbon fiber links in the form of nearly straight lines at both ends of the fracture, and the carbon fibers are stretched at A, B, C and D in the figure.

The carbon fiber is stretched lengthwise along the skeleton at point A, laterally along the skeleton at point B and diagonally in two different directions at points C and D. A and B are stretched in two perpendicular directions; therefore, the carbon fiber skeleton exhibits isotropic mechanical properties. At point A, B, C and D, there are stretched helical carbon fibers on both sides of the fracture trace and broken helical carbon fibers with corresponding structures on both sides, indicating that the helical carbon fibers will deform when subjected to tension, and it will recover partial shape variables after fracture.

In Figure 12b, a nearly straight carbon fiber passes through the middle of a broken double helical carbon fiber to form a hooked structure; the contact points are E and F. The contact points E and F are taken as the dividing line to observe the broken double helical carbon fiber. L_1_ and L_2_ show that the pitch of the left end of the dividing line is about 2.5 μm, while the pitch of the right end is 4 μm. Since L_1_ and L_2_ are not equal, there is still deformation inside the broken double helical carbon fiber. The shape of the helical carbon fiber is very variable, and it can even be straightened into a straight line. In Figure 12b, the straight carbon fiber and the helical carbon fiber together resist the tearing of the carbon fiber skeleton, which demonstrates the synergy between the carbon fibers.

## 4. Discussion

The EDS and XPS measurement results did not contain iron and tungsten elements, indicating that our sample did not contain catalytic particles. XPS measurements show that the helical carbon fiber contains C sp^3^. We believe that C sp^3^ drives the helical growth of carbon fiber, and the growth mechanism is as follows: 1. C sp^3^ has a non-planar structure; when C sp^3^ is incorporated into the graphite layer, it will cause the layer to bend, which can make the plane concave or convex, with random distribution. 2. The higher the concentration of C sp^3^ in the layer, the more intense the layer bending. When the experimental conditions are fixed, the bending angle is fixed. 3. Once a curved surface is formed, the curved surface will make C sp^3^ arranged in an orderly manner, uniform toward the concave or convex surface, which causes the carbon fiber to continue to grow along the fixed helical diameter and angle, forming a helical structure. 4. In the process of helical growth, there are fluctuations, so the ordered arrangement of C sp^3^ is broken, and with the growth, a new ordered arrangement is formed. Then, it grows in different directions with the same helical diameter. This is the change-direction ring observed in the experiment.

The experimental parameters of capacitance, resistance, inductance, and voltage were held constant in the arc glow discharge device. Under specific conditions, an increase in distance resulted in a decrease in current and an increase in discharge power within the discharge zone. However, adjusting the growth current to approximately 2.2 A caused excessive discharge power between the tungsten needle electrodes leading to melting and deformation of their heads. This created a vicious cycle where increasing distance further exacerbated instability during growth experiments.

**Figure 13:**
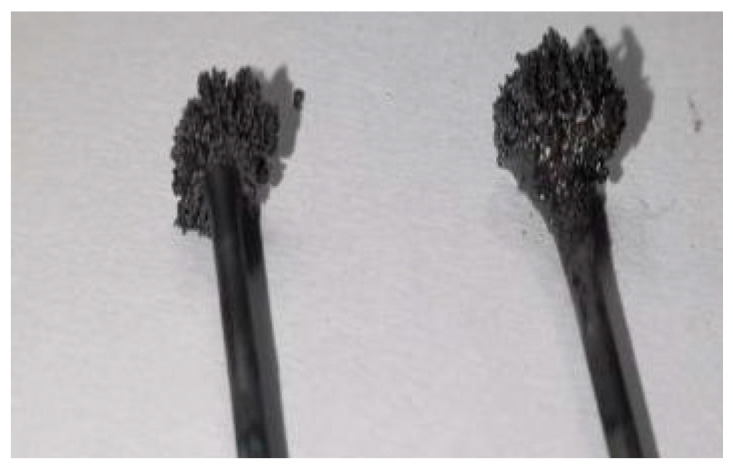
The head of tungsten needle electrode grows.

Conversely, setting the growth current at around 3.0 A led to lower discharge power that allowed for appropriate head growth on tungsten needle electrodes while also causing them to become larger over time, as shown by Figure 13. Unfortunately, this eventually resulted in the termination of experiments due to discharges occurring with graphite cavity side walls. Therefore, in order for the instrument to perform sample growth efficiently and continuously, the current size should be controlled between 2.3 A and 2.9 A.

## 5. Conclusions

The helical carbon fiber skeleton samples were prepared on iron wire substrate by the arc glow discharge method. As the current decreased, the diameter of carbon fiber increased. As the current increased, the number of tiny carbon fibers also increased. The results of EDS and XPS show that the carbon fiber skeleton did not contains Fe or W elements, but it did contain C sp^3^. A growth mechanism of helical carbon fiber driven by C sp^3^ was proposed. The appearance of a change-direction ring indicates that the growth direction of carbon fiber has changed. This often leads to the return of the original spiral growth path, forming a double helix structure. The longitudinal growth splitting mode of carbon fiber and the secondary nucleation growth mode of side wall were observed, which can greatly increase the specific surface area of helical carbon fiber.

Multiple nucleation and diverse growth made helical carbon fibers hook to each other to form a helical carbon fiber skeleton. A super-large carbon fiber with a diameter of about 13 μm was observed, which may have been the backbone of a long helical carbon fiber skeleton. The helical carbon fiber would deform when subjected to tension, and it would recover partial shape variables after fracture. There was a synergy between carbon fiber and carbon fiber inside the helical carbon fiber skeleton. The deformation of carbon fibers due to resistance existed on the cracks in different directions, which indicated that the carbon fiber skeleton had isotropic mechanical properties. The results of this paper are helpful for promoting the development of carbon fiber-reinforced composites.

## 6. Patents

System for preparing helical carbon fiber skeleton. Xiye Chen, Haiyong Chen & Zhigang Jiang, Priority No. CN202310862380.9.

## Data Availability

The original contributions presented in the study are included in the article, further inquiries can be directed to the corresponding author.
